# An Atypical Porencephalic Cyst Manifesting as a Simple Partial Seizure: A Case Report and Literature Review

**DOI:** 10.1155/2017/2174045

**Published:** 2017-09-05

**Authors:** Abdulaziz Ibrahim Al Thafar, Abdullatif Sami Al Rashed, Bayan Abdullah Al Matar, Abdulaziz Mohammad Al-Sharydah, Abdulrahman Hamad Al-Abdulwahhab, Sari Saleh Al-Suhibani

**Affiliations:** ^1^College of Medicine, King Faisal University, Al-Ahsa City, Saudi Arabia; ^2^Radiology Department, King Fahd Hospital of the University, Imam Abdulrahman Bin Faisal University (University of Dammam), Dammam, Eastern Province, Saudi Arabia

## Abstract

**Background:**

Porencephaly is an extremely rare neurological disease characterized by the presence of solitary or multiple degenerative cerebrospinal fluid (CSF) cavities within the brain parenchyma.

**Case Report:**

We describe a case involving a 23-year-old male who presented with involuntary movements of the left upper limb of 6 months' duration. A diagnosis of porencephaly was confirmed by magnetic resonance imaging (MRI).

**Conclusion:**

The rarity of occurrence and atypical presentation of such a lesion present a challenge to clinicians. Little is known about the pathogenesis and appropriate management of porencephaly. Further studies of the implications of porencephaly for neurodevelopment and behavior are needed.

## 1. Introduction

Porencephaly is an extremely rare neurological disease characterized by the presence of solitary or multiple degenerative cerebrospinal fluid (CSF) cavities within the brain matter [1]. Two types of porencephaly have been described: congenital porencephaly and acquired porencephaly [2]. Acquired porencephaly occurs because of infarction, trauma, hemorrhage, infection of the brain parenchyma, and other idiopathic causes [3]. These cavities or cysts may be mild enough to remain unnoticed or severe enough to cause physical and mental disorders. Unfortunately, no population-based studies of the prevalence of porencephaly have been conducted in Asian countries to date. Furthermore, the overall prevalence of this condition has not yet been explored [4]. A wide spectrum of porencephaly-associated clinical presentations have been reported in the literature [5–8]. To our knowledge, this is the first case of a simple partial seizure caused by an acquired porencephalic cyst, which initially presented with involuntary movements of the left upper limb.

## 2. Case Presentation

A 23-year-old Saudi male presented to the neurology clinic complaining of involuntary left upper limb movements of 6 months' duration. The movements lasted for few seconds and stopped when the arm was held by the other hand. The movements were described as supination and pronation and occurred only when the patient was experiencing stressful situations or getting insufficient sleep. No other factors appeared to induce the attack, which was limited to the left arm. There was no change in the level of consciousness during or before the attacks and there were no other associated symptoms.

The patient's medical history revealed tonic-clonic seizures at 9 years of age, which subsided by the age of 14 years. The seizures occurred during sleep, and there were approximately 6–8 episodes over those 5 years. The patient had received carbamazepine 200 mg per day and had discontinued the medication when the attacks subsided. He had no other significant medical history. The patient's birth history revealed that he was delivered at term by cesarean section, and he was placed in an incubator for 17 days after birth for unknown reasons. No further details were provided regarding his birth history. His surgical history was significant in terms of open reduction and internal fixation of a pathological fracture of the tibia and fibula after a minor sports injury; a nonossifying fibroma was the underlying cause of the pathological fracture. His family history was noncontributory. His general and systemic physical examinations were normal.

The patient's neurological assessment was significant only for hyperreflexia of the left upper limb (+3 for left brachioradialis, biceps, and triceps reflexes). The results of his motor, sensory, and cerebellar examinations were normal. The Babinski sign was negative. No abnormalities were found by hematological and biochemistry blood tests (Table 1).

### 2.1. EEG and Imaging

An EEG recording showed background activity of a well-regulated alpha rhythm, at a frequency of 9 Hz, with maximum amplitude of 70 mcv (referential). Photic stimulation induced a good driving response. Hyperventilation added no further information. Interictal EEG was normal. These findings were consistent with a normal EEG.

**Figure 1:**
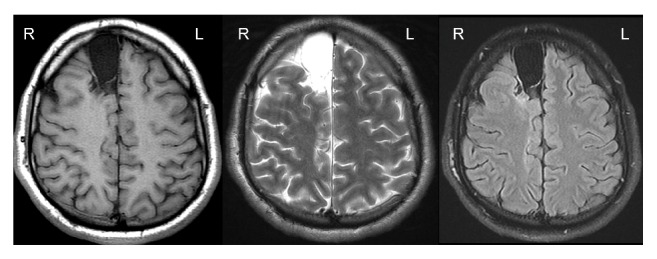
Transaxial multisequential magnetic resonance imaging of the brain in the form of a T1 weighted image (WI), T2 WI, and fluid-attenuated inversion recovery (FLAIR) WI, showing an extra-axial, well-defined elliptical lesion located in the right frontal region. The lesion follows the signal intensity of the CSF in all sequences.

**Figure 2:**
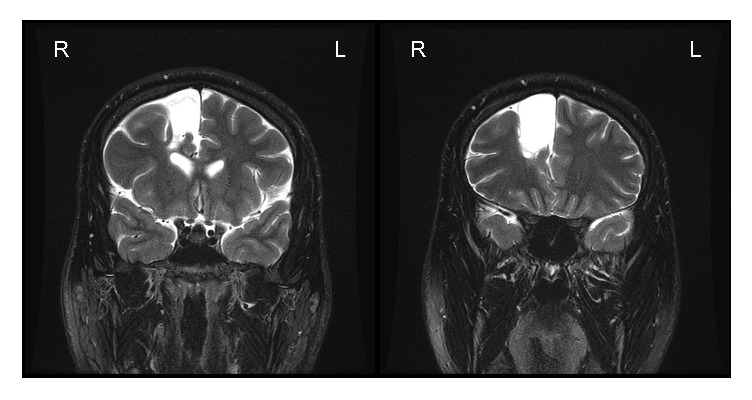
A coronal T2 fat-saturated image demonstrates extra-axial T2 signal hyperintensity of the right frontal region, which communicated with the subarachnoid space (anterior interhemispheric fissure) but had no communication with the ventricular system.

**Figure 3:**
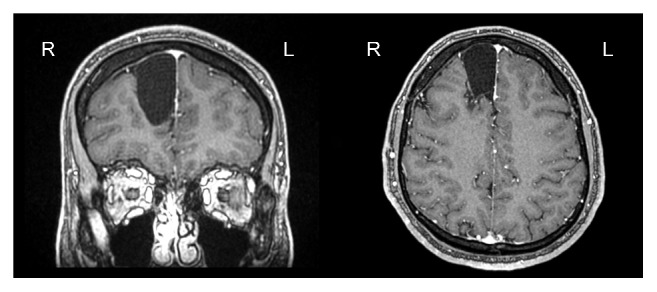
Three-dimensional fast spoiled gradient-echo (3D FSPGR) postcontrast axial and coronal images, showing no definitive enhancement of the right frontal lesion.

Brain MRI with contrast showed corticosubcortical cystic encephalomalacic changes, centered on the right superior frontal gyrus and contacting the right lateral ventricle frontal horn (Figures 1 and 2). These changes included a dominant large unilocular cystic component, measuring around 4 × 3 × 2.3 cm, which followed the CSF signal on all sequences and lacked overt hemosiderin staining. Thinning of the overlying cortical mantle was present, with mild scalloping of the adjacent calvarial inner table. The dominant cyst was surrounded by smaller cystic changes (Figure 3).

These findings were suggestive of a remote insult, probably from a posttraumatic or perinatal ischemic injury, and were consistent with a porencephalic cyst.

### 2.2. Final Diagnosis and Treatment Plan

The final diagnosis was a simple partial motor seizure caused by acquired porencephaly. Lifestyle modifications and levetiracetam 1000 mg twice daily were prescribed. A follow-up consultation was scheduled for 1 month later. The patient changed his lifestyle by avoiding major stressors and ensuring that he obtained sufficient sleep. No additional seizures were experienced while he was on the above treatment plan.

## 3. Discussion

### 3.1. Pathogenesis and Clinical Presentation

Acquired porencephaly can occur as a result of prenatal or postnatal factors, such as ischemia, traumatic brain injury, or hemorrhage. Hypoperfusion leads to focal encephalomalacia, focal necrosis of both the gray matter and white matter, and eventually cystic degeneration. The pathogenesis of the symptoms is not well understood, but many factors can trigger symptoms in patients, including stressful life events [9] and traumatic injuries [11, 12]. Symptoms can also manifest spontaneously, without any known triggers [7, 8, 10].

Many of the studies linking the onset of epilepsy with emotional events have methodological weaknesses and have failed to assess possible confounding factors prospectively [13]. Evidence has suggested that individuals with epilepsy who report experiencing emotional seizure triggers show attentional bias toward threats. This association is complicated by the fact that stressful experiences are linked to other potential seizure-precipitating factors (such as sleep deprivation). Thus, the probable indirect trigger for our patient's symptoms was a stressful life event (his father's death).

A wide range of symptoms of porencephaly have been reported in the literature. Most of the reported cases presented with their first psychotic episode before the diagnosis of porencephaly [7, 9, 10]. Our patient presented with a simple partial seizure, with no psychotic symptoms. Bhagyabati Devi et al. reported a case of porencephaly and seizures. The patient presented with multiple types of seizures, which was somewhat similar to our findings [6]. Ryzenman et al. reported a case of porencephaly in which the patient presented with spontaneous CSF otorrhea caused by a massive porencephalic cyst [8]. Another case presented with posttraumatic diplopia [11]. These variations in clinical presentation can be explained by differences in the sizes and sites of the lesions among the cases. The existing literature on patients with late presentation of acquired porencephaly is summarized in Table 1.

### 3.2. Diagnosis and MRI Findings

MRI is the gold standard for a diagnosis of porencephaly. The typical finding is a cystic space in the brain parenchyma which communicates with an enlarged adjacent ventricle [4]. The diagnostic findings in our case were unique. The MR images revealed extra-axial cystic encephalomalacic changes located on the right superior frontal gyrus (Figures 1 and 2) and abutting the right lateral ventricle frontal horn, including a dominant large unilocular cystic component roughly measuring 4 × 3 × 2.3 cm in maximum dimensions. This cyst followed the CSF signal intensity on the sequences without overt hemosiderin staining and caused thinning of the overlying cortical mantle with mild scalloping of the adjacent calvarial inner table (Figure 3). The etiology of these findings is likely a posttraumatic vascular insult, given that the patient was maintained in an incubator for 17 days postnatally for unknown reasons. However, the patient and his mother denied any vascular insult during birth. Nonetheless, an incidental note was made of a developmental venous anomaly at the temporal horn gray-white matter transition, which displayed a satisfactory age-expected appearance. The overall cerebral trophicity was satisfactory, and the hippocampi showed an acceptable anatomical configuration, trophicity, and signal intensity. No evidence of acute or subacute ischemia was seen on diffusion-weighted images.

### 3.3. Differential Diagnosis

The differential diagnosis included the following conditions: neuroglial cyst, arachnoid cyst, schizencephaly, and ependymal cyst. The diagnosis of porencephaly can easily be missed, since other intracranial cysts can mimic this condition. Neuroglial, or glioependymal, cysts are benign epithelial-lined lesions that can occur anywhere in the neuraxis. Neuroglial cysts appear well-demarcated, without surrounding gliosis, and have the same appearance as the CSF in all sequences [4]. An arachnoid cyst is a benign congenital extracerebral mass that contains CSF surrounded by the arachnoid membrane [14]. Arachnoid cysts are extra-axial and displace the brain cortex from the adjacent skull [4]. Schizencephaly is a gray matter-lined cleft that extends from the pial surface to the ventricle [15]. On imaging, schizencephalic lesions are lined with heterotopic gray matter and extend from the ventricle to the brain surface [4]. Ependymal cysts are benign cysts of the lateral ventricle or juxtaventricular area of the temporoparietal region and frontal lobe [4]. Typically, they present intraventricularly with normal neighboring brain tissue [4].

In conclusion, due to its rarity of occurrence and atypical presentation, porencephaly presents a challenge to clinicians. Little is known about the pathogenesis and treatment of this condition. Further studies are required on this and other cephalic cystic disorders. The extra-axial involvement of this lesion might require further classification in the future. Imaging is essential to establish a diagnosis and thus to determine the best treatment option.

## Figures and Tables

**Table 1:** Summary of previously reported cases of acquired porencephaly.

	Author [reference]	Age/sex	Presentation	Lesion location	Treatment
(1)	Noyan et al. (2016) [9]	43/female	First psychotic episode	Right medial frontal lobe	Antipsychotics

(2)	Hussain et al. (2015) [10]	26/female	First psychotic episode	Left side of frontal lobe	Antipsychotics

(3)	Sarmast et al. (2012) [11]	12/male	Posttraumatic diplopia	Left parietooccipital region	Cystoperitoneal shunt

(4)	Douzenis et al. (2010) [7]	25/female	First psychotic episode	Frontotemporal lobes	Antipsychotics

(5)	Ryzenman et al. (2007) [8]	65/female	Congenital hemiplegia, left-sided CSF otorrhea, and hearing loss	Left cerebral hemisphere.	Transmastoid approach

(6)	Bhagyabati Devi et al. (2002) [6]	15/male	Various seizure patterns	Left cerebral hemisphere	Anticonvulsants

(7)	Nakao et al. (1991) [12]	33/male	Posttraumatic headache	Left frontal lobe	Resection and corticotomy

(8)	Our case	23/male	Simple partial seizure	Right frontal lobe	Anticonvulsants
